# Comparison of Clinical and Imaging Parameters in Patients With Neuromyelitis Optica Spectrum Disorder and Myelin Oligodendrocyte Glycoprotein Antibody-Associated Disease: A Prospective Observational Study

**DOI:** 10.7759/cureus.89840

**Published:** 2025-08-11

**Authors:** Ekta Devi, Shishir K Chandan, Divya Rani, Dipti P Mohapatra, Shreya Verma

**Affiliations:** 1 Neurology, Vardhman Mahavir Medical College and Safdarjung Hospital, New Delhi, IND

**Keywords:** aqp4 antibody, longitudinally extensive transverse myelitis, mog antibody, myelin oligodendrocyte glycoprotein antibody disease, neuromyelitis optica, optic neuritis

## Abstract

Aim/background

The concept of neuromyelitis optica spectrum disorder (NMOSD) is changing, with a disease spectrum emerging that includes aquaporin 4 (AQP4) IgG-seropositive NMOSD, myelin oligodendrocyte glycoprotein (MOG) antibody-associated disease (MOGAD), and double-seronegative NMOSD. The past years have seen important advances in understanding rare demyelinating central nervous system (CNS) disorders associated with AQP4-IgG and MOG-IgG antibodies. Most of the recent literature has focused on the identification of clinical and magnetic resonance imaging (MRI) features that help distinguish these diseases from each other, simultaneously highlighting major diagnostic pitfalls that may lead to misdiagnosis. The present study aims to understand the epidemiology and disease characteristics of NMOSD and MOGAD in our population and compare them with previously published reports.

Materials and methods

This was a prospective, single-center, comparative, observational study conducted over 18 months. Thirty patients were recruited and categorized into two groups: NMOSD and MOGAD. Each group consisted of 15 patients. Data regarding neurological assessment, neuroimaging, treatment, and outcome were collected. These patients were followed at one, three, six, and 12 months for treatment response, residual disability, and relapse. Disease severity and disability were assessed by using the Expanded Disability Status Scale (EDSS) and modified Rankin scale (mRS).

Results

The average age at presentation of the NMOSD group of patients was 34.67 ± 15.66 years, which was significantly higher compared to the 26 ± 5.74 years seen for the MOGAD group (p < 0.0001). The MOGAD group of patients had a significantly higher proportion of men compared to the NMOSD group (66.67% in MOGAD versus 0% in NMOSD). Optic neuritis was seen in a significantly higher proportion of MOGAD patients compared to the NMOSD group of patients (p = 0.0061). Bilateral optic neuritis was more common in the MOGAD group (26.67% vs. 6.67% in the NMOSD group). Isolated myelitis was higher in the NMOSD group. A higher proportion of patients in the NMOSD group received steroids along with rituximab (26.67%) compared to the MOGAD subgroup of patients. In terms of the rescue treatment, intravenous immunoglobulin (IVIG) or plasma exchange (PLEX) therapy was required more in the NMOSD group than the MOGAD group. The EDSS and mRS scores of both groups were comparable at baseline. However, on follow-up, the EDSS and mRS levels were significantly lower for the MOGAD group compared to the NMOSD group (p < 0.05). The overall relapse rate was 33.33% in the NMOSD group compared to 20% in the MOGAD group at 12 months.

Conclusion

NMOSD and MOGAD are two distinct CNS demyelinating disorders having different demographics, clinical profiles, treatment responses, relapse rates, and short-term outcomes. MOGAD patients appear to have younger age at onset, male predominance, less severe clinical presentation, good response to first-line treatment, fewer relapses, and better one-year functional outcomes whereas NMOSD has female predominance, more severe clinical attacks of myelitis and optic neuritis, less response to first-line management of acute attack requiring rescue therapy more often, less response to conventional immunosuppressive treatment with more relapses requiring escalation of maintenance therapy with rituximab, and significant visual and locomotor residual disability at 12 months.

## Introduction

Neuroimmune disorders of the central nervous system (CNS) encompass a wide spectrum of conditions. Many of these disorders result in acquired demyelination of the brain and spinal cord. These neuroimmune demyelinating disorders manifest across the age spectrum, and the clinical phenotypes, radiologic expression, treatment, and prognostic considerations are often overlapping.

Neuromyelitis optica (NMO) is a rare and predominantly relapsing inflammatory disease that affects the CNS. For many years, NMO was considered an overlap syndrome with multiple sclerosis (MS), distinguished from it only by its relative severity and specific predilection for optic nerve and spinal cord involvement [1]. The recognition of NMO spectrum disorder (NMOSD) as an autoimmune astrocytopathy has been relatively recent after the discovery of serum autoantibodies that target the astrocytic water channel aquaporin 4 (AQP4) in 2005 [2]. Later, it was found that in 20%-30% of patients diagnosed with NMOSD, AQP4-IgG is undetectable, sparking debate on whether AQP4-IgG-seropositive and AQP4-IgG-seronegative results represent the same condition or are distinct disease entities [3]. Many case reports and series have documented the presence of serum myelin oligodendrocyte glycoprotein (MOG)-IgG in some patients with AQP4-IgG-seronegative NMOSD [4]. Later, it became clear that these antibodies were also detectable among patients with acute disseminated encephalomyelitis (ADEM), recurrent optic neuritis (ON), and autoimmune encephalitis [5]. The full clinical spectrum of MOG antibody-associated disease (MOGAD) is yet to be discovered. It is also not clear whether NMOSD and MOGAD represent a similar clinical, radiological, and treatment outcome profile or whether they are distinct disease entities that require a separate approach for diagnosis, treatment, and management. With this context, our study aimed to compare clinical and radiological parameters in patients with NMOSD and MOGAD for a better understanding of their clinical spectrum and further management.

## Materials and methods

This was a single-center, prospective, comparative, observational study conducted over 18 months. Patients 18 years and above satisfying the International Panel for NMO Diagnosis-2015 criteria for NMOSD with anti-aquaporin antibody-positive serology [6] and patients fulfilling the diagnostic criteria for MOGAD with anti-MOG antibody-positive serology [7] were enrolled in our study and were categorized into the NMOSD and MOGAD groups, respectively. Only patients diagnosed as NMOSD and MOGAD at the first clinical presentation were enrolled in the study. Cases with prior relapses, either diagnosed or previously undiagnosed, were excluded from the study. Patients lost to follow-up were also excluded from the analysis. The sample size was calculated based on prevalence in both groups taken from previous literature, with an alpha type I error of 5% and beta of 80% power. The study sample size calculated was 15 for each group.

Demographic data of all the patients were recorded. A detailed neurological examination was done. Disease severity and disability were documented in terms of the Expanded Disability Status Scale (EDSS) and modified Rankin scale (mRS). A detailed ophthalmological evaluation of all the patients was done. Both eyes of each patient were evaluated for best-corrected visual acuity and fundoscopy. All patients were given a visual functional severity score (VFSS) based on their visual acuity (rated from 0 to 6). Cerebrospinal fluid (CSF) analysis; contrast-enhanced magnetic resonance imaging (MRI) of the brain, orbit, and spine; visual evoked potential; and brainstem auditory evoked response were also recorded. Anti-aquaporin 4 antibody and anti-MOG antibodies were tested by a fixed cell-based indirect immunofluorescence assay in serum. Treatment, including both acute and maintenance therapy, and outcome details were recorded.

All the patients were followed up at one month, three months, six months, and 12 months. Lost to follow-up cases were not included in the analysis. On follow-up, detailed neurological and ophthalmological evaluations were repeated. Patients’ therapeutic responses in terms of recovery, number of relapses, change in EDSS and mRS score, and requirement of steroid-sparing agents were also recorded. At each follow-up visit, the dose, duration, and side effects of ongoing therapy were also noted. Treatment was given as per the standard treatment protocol and individualized according to the severity of illness, cost-effectiveness of the treatment, patient’s past drug history, and comorbidities.

Statistical analysis

Data were coded and recorded in the MS Excel spreadsheet program (Microsoft Corp., Redmond, WA, US). SPSS v23 (IBM Corp., Armonk, NY, US) was used for data analysis. Descriptive statistics were elaborated in the form of means/standard deviations and medians/IQRs for continuous variables and frequencies and percentages for categorical variables. Data were presented in histograms, box-and-whisker plots, and column charts for continuous data and bar charts or pie charts for categorical data. Group comparisons for continuously distributed data were made using an independent sample “t”-test when comparing two groups. If data were found to be non-normally distributed, appropriate non-parametric tests in the form of the Wilcoxon test/Kruskal-Wallis test were used for these comparisons. The Chi-squared test was used for group comparisons for categorical data. In case the expected frequency in the contingency tables was found to be <5 or >25% of the cells, Fisher’s exact test was used instead. Linear correlation between two continuous variables was explored using Pearson’s correlation (if data were normally distributed) and Spearman’s correlation (for non-normally distributed data). A p-value of <0.05 was considered statistically significant.

## Results

Demographic and clinical features

**Figure 1:**
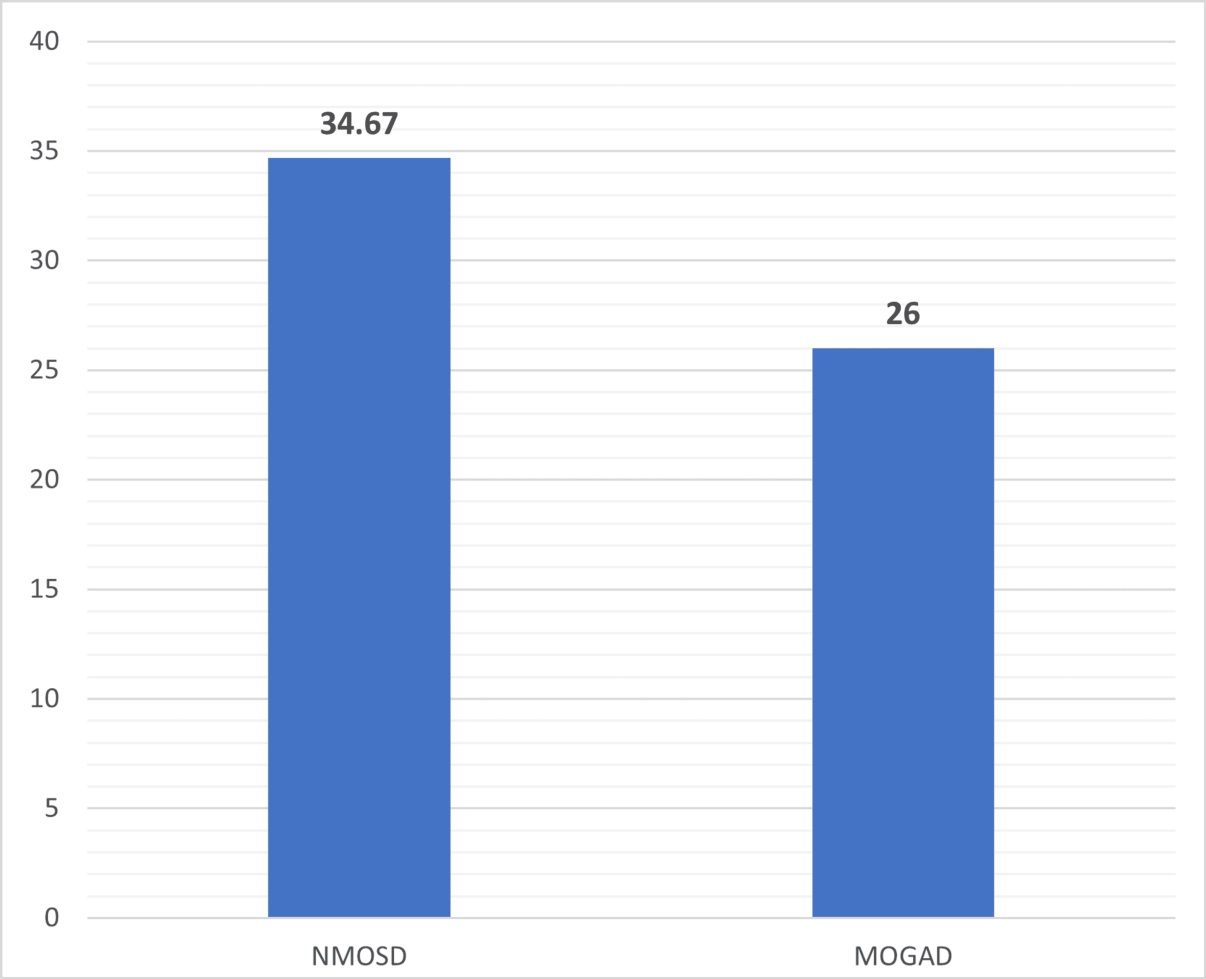
Comparison of age (years) of presentation between NMOSD and MOGAD in the study cohort NMOSD: neuromyelitis optica spectrum disorder; MOGAD: myelin oligodendrocyte glycoprotein antibody-associated disease

**Figure 2:**
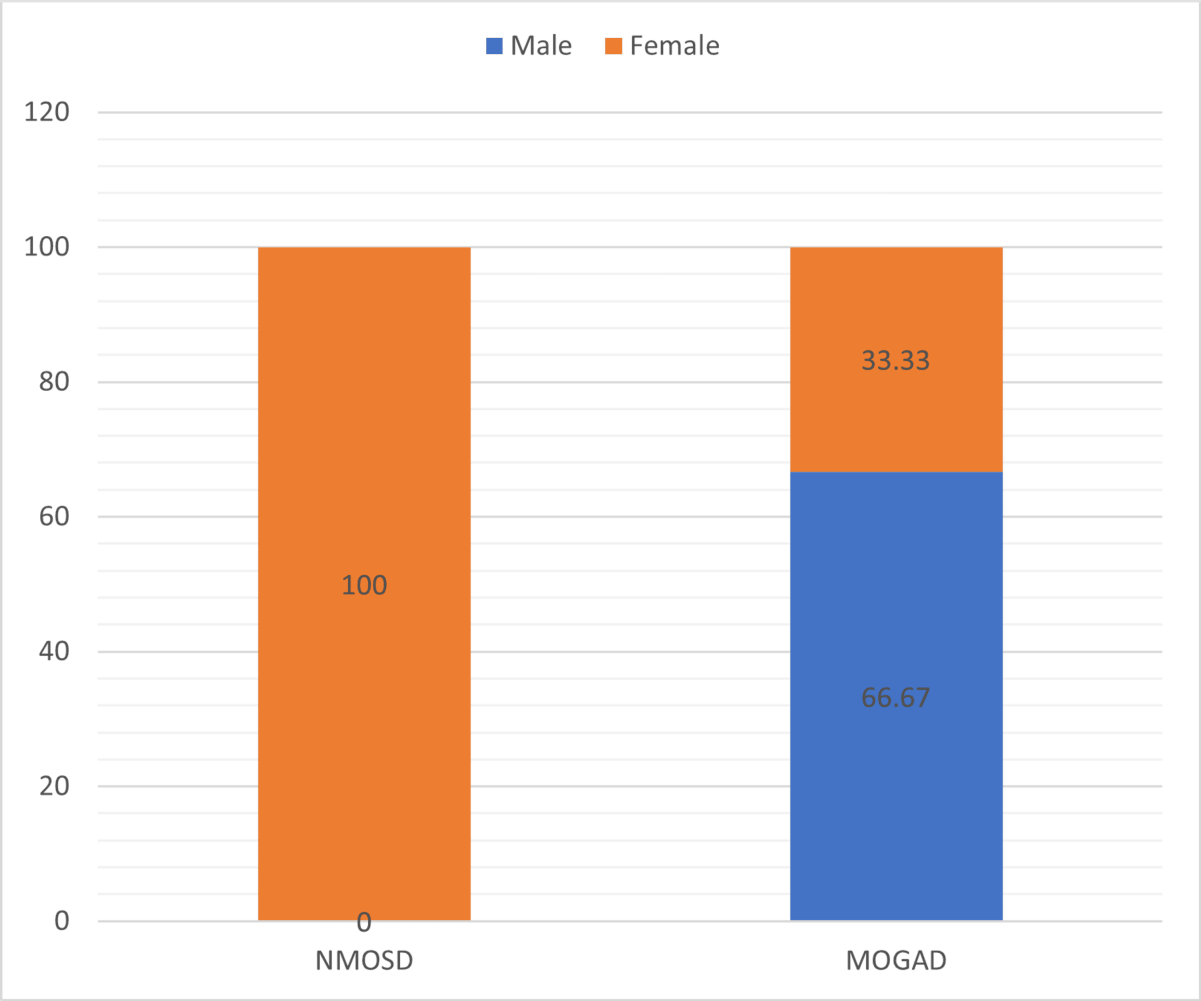
Comparison of the male and female ratio between the two groups NMOSD: neuromyelitis optica spectrum disorder; MOGAD: myelin oligodendrocyte glycoprotein antibody-associated disease

In our study, 15 patients in each group who completed the study period were taken for analysis of the results. In our study, the mean age of presentation (years) of the NMOSD group was 34.67 ± 5.66, which was significantly higher (p-value < 0.0001) compared to the MOGAD group, for which the mean age of presentation was 26 ± 5.74 (Figure 1). The MOGAD group of patients had a significantly higher proportion of men (66.67%) compared to the NMOSD group, where all patients were women, which was also statistically significant (p-value < 0.0001) (Figure 2). In the NMOSD group, myelitis was the most common presentation, while in the MOGAD group of patients, ON was the most common presentation. Myelitis was found in 14 patients (93.33%) in the NMOSD group compared to seven (46.67%) patients in the MOGAD group (p = 0.0061). The MOGAD group had a greater number of patients presenting with ON (73.34% vs. 26.67%, p = 0.0120). Both unilateral and bilateral optic nerve involvement were more common in the MOGAD group, but bilateral ON was significantly higher in the MOGAD group (26.67% vs. 6.67%, p = 0.0318). Visual acuity was severely impaired (6/60-PL positive) in all four (100%) affected cases of NMOSD, whereas only two (18.88%) out of 11 affected cases in MOGAD (p = 0.382). Our study also showed that area postrema syndrome, brainstem syndrome, encephalopathy, and focal cerebral symptoms were more commonly seen in the NMOSD group as compared to the MOGAD group. However, the difference was not statistically significant (Table 1).

**Table 1:** Demographics and clinico-radiological phenotype comparison between the NMOSD and MOGAD groups *Paired t-test NMOSD: neuromyelitis optica spectrum disorder; MOGAD: myelin oligodendrocyte glycoprotein antibody-associated disease; ON: optic neuritis; severely impaired visual acuity: vision 6/60 or less Area postrema syndrome is manifested by intractable hiccups, nausea, and vomiting. Brainstem syndromes include diplopia, bulbar dysfunction, vertigo, or ataxia. Focal cerebral syndromes include aphasia, hemiparesis, and visual field defect. Encephalopathy includes seizures and impaired consciousness

	NMOSD	MOGAD	p-value*
Total number of patients	15	15	
Median age (years)	34.67 ± 5.66	26 ± 5.74	<0.0001
Female (%)	15 (100%)	5 (33.33%)	0.0001
Myelitis (%)	14 (93.33%)	7 (46.67%)	0.0061
ON	4 (26.67%)	11 (73.34%)	0.0120
Unilateral ON (%)	3 (20%)	7 (46.67%)	0.263
Bilateral ON (%)	1 (6.67%)	4 (26.67%)	0.0318
Severely impaired visual acuity	4 (26.67%)	2 (13.33%)	0.382
Area postrema syndrome	3 (20%)	0	0.0726
Brainstem syndrome	3 (20%)	1 (6.67%)	0.2911
Encephalopathy	2 (13.33%)	0	0.1501
Focal cerebral symptoms	1 (6.67%)	0	0.0726
Multifocal presentation (myelitis with optic neuritis or myelitis with area postrema syndrome)	6 (40%)	3 (20%)	0.0726

MRI features

The spinal cord was more commonly involved in the NMOSD group (93.33% vs. 46.67%) compared to the MOGAD group (p = 0.0061). All the patients with spinal cord involvement had longitudinally extensive transverse myelitis (LETM) irrespective of the group. Cervical myelopathy was exclusively seen in the NMOSD group. The thoracic segment was more commonly involved in the MOGAD group as compared to the NMOSD group, but it was not statistically significant (0.6711). Conus involvement was seen only in one patient of the MOGAD group (6.67%). Similar to clinical presentation, optic nerve lesion was more commonly seen in the MOGAD group compared to the NMOSD group (60% vs. 26.67%). Both unilateral and bilateral optic nerve MRI lesions were more common in the MOGAD group. Long segment involvement of optic nerves was more common in the NMOSD group (26.67% vs. 13.33%) while anterior segment involvement was more commonly seen in the MOGAD group (p = 0.0149). Chiasmal involvement was seen only in one patient in the NMOSD group, whereas none of the patients in the MOGAD group had chiasmal involvement. Three patients in the NMOSD group having area postrema syndrome (20%) had a lesion in the dorsal medulla, whereas in the MOGAD group, none of the patients showed area postrema involvement. Similarly, cervicomedullary junction involvement was also more commonly seen in the NMOSD group compared to the MOGAD group (26.67% vs. 13.33%), though the results were not statistically significant. Cerebral involvement was seen to be higher in the MOGAD group (33.33% vs. 13.33%), but the p-value was not statistically significant. Brainstem, periventricular, and corpus callosal hyperintensities on MRI were equally seen in both groups. Cerebellar, diencephalon, and peri-ependymal involvement was not seen in any group (Table 2).

**Table 2:** Radiological characteristics of the study cohort *Paired t-test NMOSD: neuromyelitis optica spectrum disorder; MOGAD: myelin oligodendrocyte glycoprotein antibody-associated disease; LETM: longitudinally extensive transverse myelitis; MRI: magnetic resonance imaging

MRI features		NMOSD	MOGAD	p-value*
Spinal cord involvement	Total number (percentage) of myelitis	14 (93.33%)	7 (46.67%)	0.0061
	LETM	14 (100%)	7 (100%)	-
	Cervical segment	5 (33.33%)	0	0.0160
	Thoracic segment	3 (20%)	4 (26.67%)	0.6711
	Conus	0	1 (6.67%)	0.3172
Optic nerve involvement	Total number (percentage) of optic nerve involvement	4 (26.67%)	9 (60%)	0.2139
	Unilateral	3 (20%)	6 (40%)	0.2399
	Bilateral	1 (6.67%)	3 (20%)	0.2911
	Anterior	1 (6.67%)	7 (46.67%)	0.0149
	Long segment	4 (26.67%)	2 (13.33%)	0.3692
	Chiasmal	1 (6.67%)	0	0.3172
Brain	Area postrema	3 (20%)	0	0.0726
	Cerebral lesions	2 (13.33%)	5 (33.33%)	0.2029
	Periventricular lesions	2 (13.33%)	2 (13.33%)	-
	Brainstem lesions	3 (20%)	3 (20%)	-
	Cervicomedullary junction involvement	4 (26.67%)	2 (13.33%)	0.3692

Treatment parameters

For acute therapy, intravenous (IV) methylprednisolone (MPS) was the most commonly used treatment agent in both groups (93.33% vs. 100%, p = 0.3172), and for rescue therapy, IV immunoglobulin (IVIG) or plasma exchange (PLEX) was used. More patients in the NMOSD group required rescue therapy than in the MOGAD group, but there was no significant difference seen between the two groups receiving either IVIG or PLEX (p = 0.326). For maintenance therapy, steroid was used either alone or with some other immunosuppressant agent (rituximab, azathioprine, or mycophenolate mofetil (MMF)). Significantly more cases in the NMOSD group were on maintenance rituximab treatment than in the MOGAD group (26.67% vs. 0%, p < 0.0347) (Table 3).

**Table 3:** Treatment parameters *Paired t-test NMOSD: neuromyelitis optica spectrum disorder; MOGAD: myelin oligodendrocyte glycoprotein antibody-associated disease; IV MPS: intravenous methylprednisolone; IVIG: intravenous immunoglobulin; PLEX: plasma exchange; MMF: mycophenolate mofetil

	Treatment parameter	NMOSD	MOGAD	p-value*
Acute therapy	IV MPS	14 (93.33%)	15 (100%)	0.3172
Rescue therapy	IVIG or PLEX	4 (26.67%)	1 (6.67%)	0.326
Maintenance therapy	Steroid only	2 (13.33%)	0	0.1501
	Steroid and rituximab	4 (26.67%)	0	0.0347
	Steroid and azathioprine	4 (26.67%)	8 (53.33%)	0.1428
	Steroid and MMF	1 (6.67%)	3 (20%)	0.2911

Recovery pattern over time

All cases were followed over time at one, three, six, and 12 months. ON recovered very fast in the MOGAD group, showing significant recovery in visual acuity of more than 6/36 at one month, and it persisted throughout the observation period of 12 months. Meanwhile, out of four cases of ON in the NMOSD group, only one showed significant recovery starting at three months. Three patients remained visually impaired at 12 months. Myelitis recovery was gradual in both groups, starting with partial recovery at one month, but all patients in the MOGAD group had significant recovery at six months. In the NMOSD group, six patients remained disabled with only partial recovery even at 12 months (Table 4).

**Table 4:** Recovery pattern over time *Independent sample t-test (NMOSD vs. MOGAD) NMOSD: neuromyelitis optica spectrum disorder; MOGAD: myelin oligodendrocyte glycoprotein antibody-associated disease Significant motor recovery: can walk without support; partial motor recovery: can walk with support; significant visual recovery: visual acuity equal to or more than 6/12 or more than 2 scale improvement; no or minimal visual recovery: visual acuity: 6/60 or less

Motor function	At 1 month			At 3 months			At 6 months			At 12 months		
	NMOSD	MOGAD	p-value	NMOSD	MOGAD	p-value	NMOSD	MOGAD	p-value	NMOSD	MOGAD	p-value*
Significant	0	0	0.8551	3	3	0.303	4	6	0.016	8	6	0.034
Partial	12	6		11	3		10	0		6	0	
No recovery	2	0		0	0		0	0		0	0	
Visual												
No or minimal improvement	4 (100%)	0	<0.0001	3	0	<0.0001	3	0	<0.0001	3	0	<0.0001
Significant improvement	0	11		1	11		1	11		1	11	

EDSS and mRS trend

**Figure 3:**
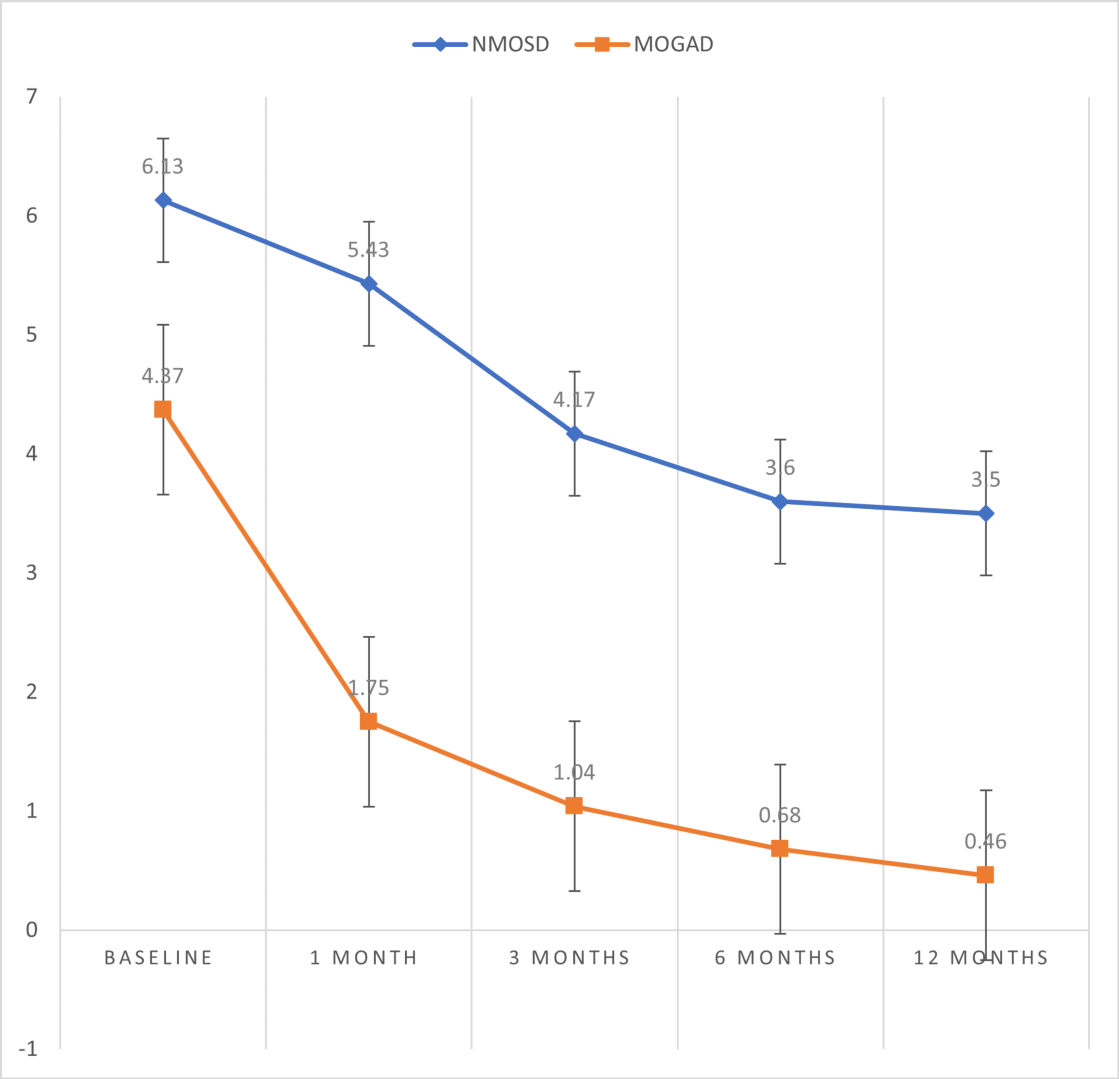
Comparison of EDSS scores EDSS: Expanded Disability Status Scale; NMOSD: neuromyelitis optica spectrum disorder; MOGAD: myelin oligodendrocyte glycoprotein antibody-associated disease

In our study, the baseline EDSS score was comparable for the two groups at baseline. But at the one-month, three-month, six-month, and 12-month follow-up time periods, the EDSS levels were significantly lower in the MOGAD group compared to the NMOSD group (p < 0.05), though the decline in the EDSS score at the 12-month follow-up in both groups was also statistically significant (p < 0.05) (Table 5, Figure 3).

**Table 5:** Comparison of EDSS at baseline and at follow-ups between the NMOSD and MOGAD groups *Paired t-test EDSS: Expanded Disability Status Scale; NMOSD: neuromyelitis optica spectrum disorder; MOGAD: myelin oligodendrocyte glycoprotein antibody-associated disease

EDSS	NMOSD	MOGAD	p-value*
Admission	6.13 ± 5.59	4.37 ± 2.07	0.2625
1 month	5.43 ± 3.11	1.75 ± 1.78	0.0004
3 months	4.17 ± 2.68	1.04 ± 1.13	0.0001
6 months	3.60 ± 2.60	0.68 ± 1.08	0.0001
12 months	3.50 ± 2.98	0.46 ± 0.89	0.0001

**Figure 4:**
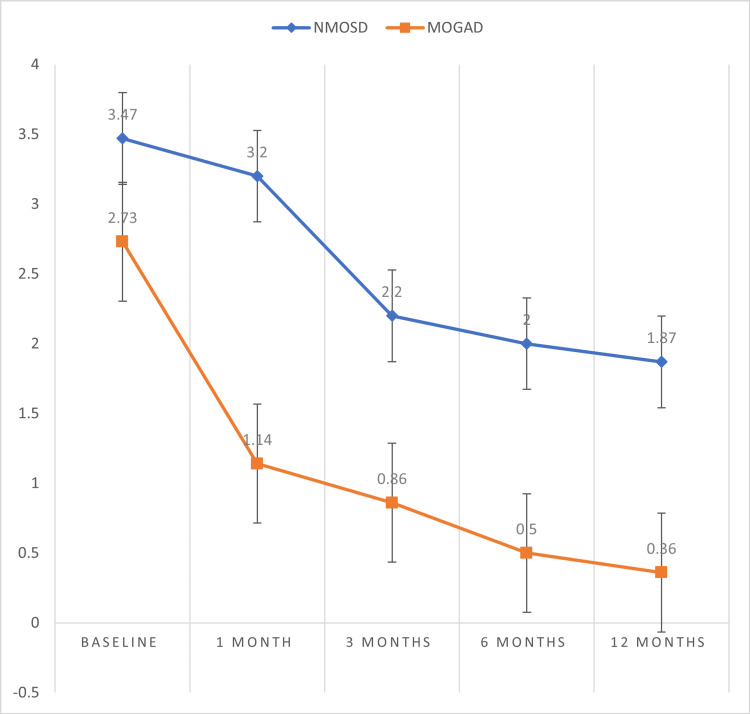
mRS trend over time mRS: modified Rankin scale; NMOSD: neuromyelitis optica spectrum disorder; MOGAD: myelin oligodendrocyte glycoprotein antibody-associated disease

Like the EDSS score, the baseline mRS score was also comparable for the two groups. On follow-up, at the one-month, three-month, six-month, and 12-month periods, the mRS levels were significantly lower for the MOGAD groups compared to the NMOSD group (p < 0.05), and the decrease in the mRS score across both groups was also statistically significant (p < 0.05) (Table 6, Figure 4).

**Table 6:** Comparison of mRS at baseline and at follow-ups between the NMOSD and MOGAD groups *Paired t-test mRS: modified Rankin scale; NMOSD: neuromyelitis optica spectrum disorder; MOGAD: myelin oligodendrocyte glycoprotein antibody-associated disease

mRS	NMOSD	MOGAD	p-value*
Admission	3.47 ± 1.73	2.73 ± 1.22	0.1866
1 month	3.20 ± 1.86	1.14 ± 1.10	0.0010
3 months	2.20 ± 1.52	0.86 ± 0.86	0.0060
6 months	2 ± 1.51	0.50 ± 0.76	0.0019
12 months	1.87 ± 1.85	0.36 ± 0.63	0.0057

**Figure 5:**
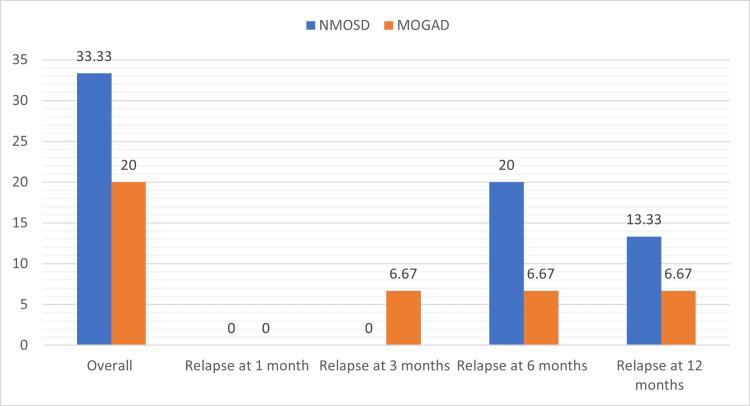
Comparison of relapse rates between the NMOSD and MOGAD groups NMOSD: neuromyelitis optica spectrum disorder; MOGAD: myelin oligodendrocyte glycoprotein antibody-associated disease

The overall relapse rate at the 12-month follow-up was 33.33% in the NMOSD group compared to 20% in the MOGAD group, which was not statistically significant (p-value = 0.4170). The earliest relapse in the MOGAD group was seen at three months, while in the NMOSD group, it was observed at six months (Table 7, Figure 5). All relapses in both groups were related to poor drug compliance.

**Table 7:** Comparison of relapse rates between the two groups *Paired t-test NMOSD: neuromyelitis optica spectrum disorder; MOGAD: myelin oligodendrocyte glycoprotein antibody-associated disease

Relapse	NMOSD	MOGAD	p-value*
Overall	5 (33.33%)	3 (20%)	0.4170
Relapse at 1 month	0	0	-
Relapse between 1 and 3 months	0	1 (6.67%)	0.3172
Relapse between 3 and 6 months	3 (20%)	1 (6.67%)	0.2911
Relapses between 6 and 12 months	2 (13.33%)	1 (6.67%)	0.5500

## Discussion

Clinical presentation

In our study, the MOGAD group of patients had a younger age of presentation. The mean age of presentation (years) of the MOGAD group was 26 ± 5.74, which was significantly lower compared to the 34.67 ± 15.66 seen for the NMOSD group (p < 0.0001). Jarius et al. and Alqwaifly et al. have also found in their study that the average age of onset for NMOSD is around 40 years, compared with the 20-30 years for MS [8,9]. Several studies have shown that MOGAD can manifest at any age but often emerges between 20 and 40 years of age, which is younger than the typical age range for AQP4-IgG-seropositive NMOSD onset [10-12]. Our study showed that all patients in the NMOSD group were women compared to the MOGAD group, where a slight male predominance was noted (p-value < 0.0001). Papp et al. and Arnett et al. noted that around 80%-90% of people with NMOSD are women [13,14]. Our results are also consistent with these major studies. In our study, ON was seen in a significantly higher proportion of MOGAD patients compared to the NMOSD group (p = 0.0120), and bilateral ON was also higher in the MOGAD group (p = 0.0318). Misu et al. found bilateral ON is more frequently seen at onset in MOGAD (31%-58%) than in AQP4-IgG-seropositive NMOSD (13%-37%) [15]. In our study, myelitis was a finding in only seven patients (46.67%) in the MOGAD group compared to 14 patients (93.33%) in the NMOSD group. Myelitis in NMOSD usually affects the cervical and upper spinal cord, whereas in MOGAD, myelitis typically affects the lower thoracic to lumbar spinal cord and conus. In both groups, cord involvement was invariably long segment (LETM). Barhate et al. and Uribe-San Martin et al. found similar findings of both NMOSD and MOGAD causing longitudinally extended lesions [16,17]. In our study, area postrema syndrome was exclusively seen in the NMOSD group. Jain et al. noted a similar finding that area postrema syndrome, manifested by intractable hiccups, nausea, and vomiting, is a characteristic brainstem syndrome in NMOSD [18]. Brainstem, encephalopathy, and focal cerebral symptoms were seen to be higher in the NMOSD group. The differences were, however, not statistically significant. These findings suggested that the NMOSD group was associated with a higher involvement of multiple brain areas. It was also observed that multifocal clinical presentation was more common in NMOSD (40%) as compared to MOGAD patients (20%), the most common being ON with myelitis and area postrema syndrome with myelitis.

MRI findings

In terms of MRI findings, our study shows that both the NMOSD and MOGAD groups had LETM in all affected patients. The NMOSD group of patients had significantly higher involvement of the spinal cord (93.33% vs. 46.67%, p = 0.0061). The NMOSD group also had significantly higher involvement of the cervical cord (p = 0.0610). Conus involvement was not seen in NMOSD patients, while one patient in MOGAD had conus involvement, but this was non-significant (p = 0.3172). Cervicomedullary junction involvement was also more commonly seen in the NMOSD group compared to the MOGAD group (26.67% vs. 13.33%), but this was also non-significant (p = 0.3692). In our study, the MOGAD group of patients was seen to have significantly higher involvement of the optic nerve (60% vs. 26.67%), but this was not significant (p = 0.2129). Bilateral optic nerve lesions were seen in 20% of MOG patients compared to 6.67% of the NMO group, and this was also non-significant (p = 0.2911). Long segment optic nerve involvement was seen to be higher in the NMO group (26.67% vs. 13.33%) while anterior segment involvement was significantly higher in the MOG group (p = 0.0149). In terms of the involvement of the brain regions, it was seen that the NMOSD group of patients had a higher rate of area postrema (20% vs. 0%) while MOGAD had higher cerebral lesions. However, these differences were non-significant. Barhate et al. showed in their study on NMO patients that all myelitis patients had LETM; 32.5% of the patients had cervicomedullary involvement [16]. According to the research by Uribe-San Martin et al., LETM was the most common MRI lesion (75%) in NMOSD [17]. In another similar study, Jain et al. [18] revealed that myelitis was the most frequent clinical characteristic to develop in patients with AQP4 antibody positivity, followed by ON, ON occurring concurrently with myelitis, and brainstem syndrome. According to Wingerchuk et al., the posterior portion of the optic nerves and the optic chiasma are predominantly affected in NMOSD [19].

Treatment modalities and outcomes

For acute treatment, MPS was the first-line agent used in both groups (93.33% vs. 100%, p = 0.3172). In terms of rescue treatment, IVIG or PLEX therapy was required more in the NMOSD group than the MOGAD group, signifying more severe disease and resistance to conventional high-dose acute steroid therapy in NMOSD, though the difference was not statistically significant (p = 0.1485). Whittam et al. found that NMOSD attacks are often severe and can result in residual deficits, particularly in people with myelitis and bilateral ON. Escalation therapy or early initiation of plasmapheresis might improve outcomes in such cases [20]. Uzawa et al. noted that PLEX is frequently needed in NMOSD, but only occasionally in MOGAD, as most people with this condition respond to IV MPS [21]. A higher proportion of patients in the NMOSD group required steroids along with rituximab (26.67%) as maintenance therapy compared to the MOGAD subgroup of patients. The difference was statistically significant, with a p-value of 0.0347. This signifies a higher failure rate of conventional immunotherapy in preventing attacks in the NMOSD group than in the MOGAD group. Whittam et al. also noted that the overall prognosis and recovery from attacks are generally better in MOGAD than in AQP4-IgG-seropositive NMOSD, with approximately 50% of people experiencing a monophasic course; therefore, many experts are cautious about starting preventive immunotherapy after the first attack in MOGAD [20].

Recovery pattern

Our study also shows that the NMOSD group of cases has severe disease at onset and poor response to treatment and is left with significant visual and locomotor disability at 12 months, whereas the MOGAD group of patients was very responsive to treatment and all patients showed early and full recovery.

EDSS and mRS trends

Both EDSS and mRS scores were comparable at baseline for both groups, but at the one-month, three-month, six-month, and 12-month follow-up period, both EDSS and mRS scores were significantly lower for the MOGAD groups compared to the NMOSD group (p < 0.05), whereas the decrease in these scores across both groups was statistically significant (p < 0.05). This suggests that a one-year functional outcome is better in the MOGAD group compared to the NMOSD group. Jagtap et al. showed that all patients of NMOSD had satisfactory outcomes (mRS, <3) [22]. Sachdeva et al. found that the functional outcome was good for about 88.9% of patients in NMOSD [23]. Zhou et al. found that one to six months after onset, the MOG-Ab+ON group showed significantly better visual function (p < 0.05) than the NMOSD [24].

The overall relapse rate at 12 months was 33.33% in the NMOSD group compared to 20% in the MOGAD group, which was non-significant (p-value = 0.4170). Myelitis was the most common presenting feature of relapse in MOGAD compared to multifocal relapse in the NMOSD group. Jarius et al. found that most patients with NMOSD experience a relapsing disease course [25]. Our results are also consistent with previous studies suggesting a higher relapse rate in NMOSD.

Strengths of this study

This study was prospective in nature, and only serologically confirmed cases were taken. Uncertain diagnostic categories like double-negative cases were excluded. Only cases with the first clinical presentation were chosen so that outcomes were accurately determined.

Limitations of the study

This study had a small sample size. As NMOSD and MOGAD are rare diseases, a larger sample size within the study period was not feasible. Treatment variability may be a potential confounder. Being a tertiary care hospital, referral bias cannot be ruled out.

## Conclusions

Our study concluded that MOGAD cases were younger, with a higher proportion of men as compared to NMOSD. ON, especially bilateral and anterior, was the most common presentation in the MOGAD group, whereas myelitis affecting the cervical cord and cervicomedullary junction was the most common presentation in the NMOSD group. Area postrema, brainstem, and cortical involvement were exclusive or more frequent in the NMOSD group. MOGAD cases responded well to first-line treatment, rarely needed IVIG or PLEX, and showed rapid, near-complete recovery with lower EDSS and mRS scores at 12 months. In contrast to that, NMOSD cases had poorer response to initial treatment, often required rescue therapy, and had higher disability at 12 months with frequent relapses, necessitating potent maintenance immunosuppression. These findings align with existing literature and add valuable insight. Larger prospective studies are still needed to deepen the understanding of these neuroimmune disorders.
